# “Putting on My Best Normal”: Social Camouflaging in Adults with Autism Spectrum Conditions

**DOI:** 10.1007/s10803-017-3166-5

**Published:** 2017-05-19

**Authors:** Laura Hull, K. V. Petrides, Carrie Allison, Paula Smith, Simon Baron-Cohen, Meng-Chuan Lai, William Mandy

**Affiliations:** 1 Research Department of Clinical, Educational & Health Psychology, University College London, London, UK; 2 London Psychometric Laboratory, University College London, London, UK; 3 Autism Research Centre, Department of Psychiatry, University of Cambridge, Cambridge, UK; 4 Child and Youth Mental Health Collaborative at the Centre for Addiction and Mental Health and The Hospital for Sick Children, Department of Psychiatry, University of Toronto, Toronto, ON Canada; 5 Department of Psychiatry, National Taiwan University Hospital and College of Medicine, Taipei, Taiwan; 6 Department of Psychology, University College London, 26 Bedford Way, London, WC1H 0AP UK

**Keywords:** Autism, Camouflaging, Coping, Sex, Gender, Social adapation

## Abstract

Camouflaging of autistic characteristics in social situations is hypothesised as a common social coping strategy for adults with autism spectrum conditions (ASC). Camouflaging may impact diagnosis, quality of life, and long-term outcomes, but little is known about it. This qualitative study examined camouflaging experiences in 92 adults with ASC, with questions focusing on the nature, motivations, and consequences of camouflaging. Thematic analysis was used to identify key elements of camouflaging, which informed development of a three-stage model of the camouflaging process. First, motivations for camouflaging included fitting in and increasing connections with others. Second, camouflaging itself comprised a combination of masking and compensation techniques. Third, short- and long-term consequences of camouflaging included exhaustion, challenging stereotypes, and threats to self-perception.

**Electronic supplementary material:**

The online version of this article (doi:10.1007/s10803-017-3166-5) contains supplementary material, which is available to authorized users.

## Introduction

Autism Spectrum Conditions (ASC)^1^ are atypical developmental conditions characterised by impairments in social interaction and communication, alongside unusually restricted/repetitive behaviours and interests, need for sameness, and atypical sensory processing (APA 2013). ASC is generally viewed as dimensional, with traits found amongst the general population and a specified cut-off point, when present with concurrent functional impairments, used to identify the clinical diagnosis (Baron-Cohen et al. 2001; Constantino 2011). One behaviour associated with ASC that has recently attracted interest is the development of camouflaging or coping strategies for use in social situations (Attwood 2007; Gould and Ashton-Smith 2011; Kopp and Gillberg 2011; Lai et al. 2011; Wing 1981). These strategies may include hiding behaviours associated with their ASC, using explicit techniques to appear socially competent, and finding ways to prevent others from seeing their social difficulties. In this paper we will refer to these behaviours as ‘camouflaging’.

While many neurotypical people, of all genders, manage the way others perceive them in social situations (Izuma et al. 2011), research suggests that individuals with ASC have a reduced ability to do so (Cage et al. 2013). However, the research in this area has focused on the manipulation of typical social behaviours, rather than how individuals with ASC may want and be able to adapt their ASC-related characteristics. Camouflaging is likely to exist on a spectrum (similar to autistic traits) in those who have an ASC diagnosis and those who are subclinical. However, self-reported evidence suggests possible categorical differences between autistic and non-autistic camouflaging. For instance, camouflaging by ASC individuals has been reported as extremely effortful and challenging to one’s identity (Bargiela et al. 2016), unlike ordinary reputation management in typically developing individuals.

Camouflaging has also been proposed as an explanation for the missed or late diagnosis of females with ASC, as part of the female phenotype or behavioural presentation (Gould and Ashton-Smith 2011; Kirkovski et al. 2013; Lai et al. 2015). Amongst clinical samples, male to female gender ratios for ASC diagnosis are generally around 4:1 (Fombonne 2009), but when active case ascertainment is used within the general population, the ratio lowers to around 3:1 (Sun et al. 2014). This discrepancy suggests that there are biases that work against females with ASC receiving accurate, timely diagnoses from clinical services. Females are less likely to receive a diagnosis of ASC than males with similar levels of autistic traits (Dworzynski et al. 2012; Russell et al. 2011), and those who receive a diagnosis on average are more likely than males receiving the same diagnosis to be older and have more additional needs, including increased intellectual disability (Shattuck et al. 2009) and behavioural-emotional challenges (Duvekot et al. 2016). Clinical experience suggests that females with ASC may be more likely than males with ASC to have been previously misdiagnosed with other mental health conditions, such as personality disorders or eating disorders (Lai and Baron-Cohen 2015; Mandy and Tchanturia 2015).

In addition to camouflaging, there are other gender differences in autistic characteristics which may contribute to late diagnosis or misdiagnosis of females. While few significant quantitative sex differences in the core symptoms have been found (Hull et al. 2016; Lai et al. 2015; Mandy et al. 2012; Van Wijngaarden-Cremers et al. 2014), comparisons of associated characteristics have shown differences between the female and male presentations (Kreiser and White 2014; Rivet and Matson 2011). For instance, males with ASC are more likely to experience externalising difficulties such as hyperactivity and conduct problems, whereas females with ASC are more likely to experience internalising problems such as anxiety and depression (May et al. 2012; Oswald et al. 2016).

These ‘qualitative’ differences between male and female presentation, including camouflaging behaviours, need to be included in measures used to assess ASC, as sex differences at a nosological level are likely to have an impact on diagnosis (Lai et al. 2015). Current diagnostic practices focus on the core ASC characteristics that have been historically established from the behavioural presentation in males, and so do not necessarily reflect the areas in which females with ASC may display different behaviours to males. As a result, current assessments of females with ASC are restricted to the areas in which females are most similar to males, and those females who do not meet the male-typical behavioural descriptions are likely to be missed (Van Wijngaarden-Cremers et al. 2014). Diagnostic biases may lead to biased sampling in studies of sex differences in ASC, such that only male-typical ASC behaviours are expected, and therefore only these behaviours are found when looked for. It has hence been argued that diagnostic assessments of ASC should include female-typical behaviours to more accurately assess ASC prevalence and characteristics across genders (Kreiser and White 2014).

Camouflaging in certain settings may lead to the perception that individuals function well and do not experience any problems, even though those individuals still experience difficulties as a result of the interaction of their ASC and the context. For example, it is suggested that girls with ASC may mimic other socially successful individuals to give the impression that they too are socially successful, but when placed in unknown environments they are not prepared for, they struggle to socialise (Attwood 2006). This may reflect both a stronger motivation to mimic, and itself be the result of a stronger motivation to ‘systemize’ social behaviour, than is seen in males with ASC. Teachers or clinicians may therefore be unaware of the difficulties being faced by girls and women with ASC, whereas family members may see their loved one in a range of situations and so realise the extent of their difficulties. Alternatively, women who receive an ASC diagnosis later on in life may have spent years feeling different and attempting to minimise this difference, until their children receive a diagnosis and they recognise the symptoms within themselves (Holliday Willey 2015).

There is a variety of anecdotal evidence of camouflaging amongst women with ASC. For instance, Liane Holliday Willey describes how she spent her life pre-diagnosis ‘pretending to be normal’, yet knowing that something was different about her (Holliday Willey 2015). In case studies of girls with ASC, researchers have suggested that the use of social imitation strategies may lead to missed, late, or questioned diagnoses (Kopp and Gillberg 1992). Essentially, social imitation may be a form of acting, whereby girls with undiagnosed ASC may be coping without receiving a diagnosis or even needing a diagnosis because their acting is relatively successful. Success here may be defined as simply not having overt functional impairments or raising concerns of teachers or other professionals, even though under the surface or behind maintaining such appearances, females may report high levels of subjective stress, anxiety and exhaustion, and a need to withdraw from social interaction to ‘re-set’. These observations have not yet been systematically tested, despite extensive interest in gender differences in ASC and the female phenotype (Gould and Ashton-Smith 2011; Kopp and Gillberg 1992; Lai et al. 2015; Robinson et al. 2013).

Individuals with ASC also display significant variation in their outcomes across the lifespan, especially concerning their social functioning. Some adults with ASC form friendships and relationships, and have fulfilling careers that enable them to remain independent (Farley et al. 2009; Strunz et al. 2016). Others, however, struggle to maintain social relationships and may remain unemployed, despite having the motivations and capabilities to work (Baldwin and Costley 2015; Shattuck et al. 2012). While some of this variation is due to individual differences in cognitive abilities, language ability, and personal preference (Howlin et al. 2000; Shattuck et al. 2012; Van Bourgondien et al. 1997), it is possible that an individual’s ability to camouflage their ASC contributes to them achieving socially desirable outcomes. Individuals who are better able to camouflage their ASC characteristics might feel more able to make friends, improve their social support, and perform better in job interviews.

However, many individuals with ASC also report extensive anxiety and depression, especially those with average-to-high levels of IQ and language abilities (Lugnegård et al. 2011). Anecdotal evidence suggests that an individual’s camouflaging can impact their mental health (Holliday-Willey 2015). Where camouflaging is unsuccessful, strenuous, or if the person feels forced to camouflage, it may be associated with high stress level, low mood and low self-esteem. In addition, the pressure to maintain successful camouflaging may lead to anxiety for individuals with ASC. Camouflaging is not necessarily a beneficial behaviour, and should not be regularly expected or encouraged for individuals with ASC, as this may risk increasing mental health problems. It is therefore important to study camouflaging in order to better understand the individual differences predicting long-term wellbeing and outcomes for individuals on the autism spectrum.

A small number of studies have recently emerged which directly examine social camouflaging behaviours in individuals with ASC. Tierney, Burns, and Kilbey (2016) interviewed ten adolescent girls with ASC about their experiences of camouflaging, and revealed some common themes including the uncertain, exhausting nature of the social environment; the desire to make friends which motivated camouflaging attempts; and using explicit techniques to mask ASC-related difficulties. Similar themes were also found during qualitative interviews with late-diagnosed women with ASC (Bargiela et al. 2016). In particular, the idea of pretending to be normal, which could be achieved through both learned and automatic strategies, and the extensive costs of such strategies, were identified. Recently, some empirical operationalisation of camouflaging behaviours in both children and adults with ASC has also been developed. Behavioural observations suggest that girls camouflage their social difficulties (e.g. by staying in close proximity to peers and weaving in and out of activities) to a greater extent on the playground than boys, and therefore are less likely to be identified as struggling socially (Dean et al. 2016). Camouflaging, operationalised as the discrepancy between (a) interpersonal behavioural presentation and (b) self-reported autistic traits and objectively measured social cognitive abilities, was found to be on-average higher in women with ASC than in men with ASC, although was associated with more symptoms of depression in men (Lai et al. 2016). These important initial studies suggest that camouflaging is a real and meaningful experience in the lives of people with ASC, and directly impacts on their social functioning and mental wellbeing.

Despite these encouraging first steps, key questions about camouflaging still need to be answered, such as how common camouflaging is within the ASC population, whether it varies across the lifetime, and whether individual differences in camouflaging are related to long-term outcomes in functioning, achievement and quality of life. In addition, the majority of those diagnosed with ASC identify as male, and a significant number of ASC individuals experience non-binary gender identities (Glidden et al. 2016; Kim et al. 2011). It is therefore important to examine camouflaging behaviours across all genders, as research so far has focused on female experiences.

Most importantly, studies of camouflaging in ASC cannot progress until a conceptual model of camouflaging has been produced, so that subsequent research has strong theoretical grounding. Such a model is best developed from a qualitative analysis of the camouflaging experiences of individuals with ASC. This will ensure that the construct of camouflaging reflects the real-life experiences of individuals with ASC rather than the preconceptions of researchers or clinicians, and that our understanding of camouflaging is representative of a broad range of individuals with ASC. Inductive (i.e. data-driven) research resulting in a comprehensive model of the camouflaging process will enable hypothesis generation and form the basis of measurement development to further explore camouflaging quantitatively.

The present qualitative study examined camouflaging in a large sample of adults of all self-identified genders who had been diagnosed with ASC, using internet-based survey and thematic analysis. Emphasis was placed on the motivations for camouflaging, techniques used, the impact that camouflaging has for the individual, and their overall attitudes to camouflaging. The aim of the study was to derive a conceptual model of camouflaging to inform future research.

The following research questions were addressed:

1. What is camouflaging?
2. What are the techniques used and what do people with ASC think camouflaging is?
3. Why do people camouflage their ASC?
4. What are the consequences of camouflaging?

## Methods

### Participants

Participants were 92 adults of 15 different nationalities (55% British). They were eligible to take part in the study if they were over the age of 16 years and had received a DSM-IV or DSM-V diagnosis from a psychiatrist or clinical psychologist in a recognized specialist clinic of an ASC, including Autism/Autistic Disorder, Asperger Syndrome/Asperger’s Disorder, Autism Spectrum Disorder, Atypical Autism, and Pervasive Development Disorder Not Otherwise Specified. Participants were recruited via the Cambridge Autism Research Database (CARD) and through adverts placed on social media. Whilst it was not possible for this study to independently verify the diagnostic status of participants, several measures were taken to check diagnostic status and establish the generalisability of findings from this sample. Participants were asked to report whether they had received an ASC diagnosis (and if so, at what age and from which type of healthcare professional) or whether they were self-diagnosed. Those who reported self-diagnosis, or who reported receiving an ASC diagnosis from someone other than a medical professional, clinical psychologist, or healthcare team, were excluded from current analysis (n = 3). Demographic characteristics of participants are included in Table 1. Participants were asked to identify their gender as ‘female’, ‘male’ or ‘other’, and give more details if they wished.

**Table 1:** Demographic characteristics of participants and whether they reported camouflaging

	Female	Male	Other gender
N	55	30	7
Age (mean years)	40.71 (SD 14.14)	48.03 (SD 16.62)	40.71 (SD 14.29)
Age (range)	18–68	22–79	27–69
Age at diagnosis (mean years)	36.98 (SD 14.21)	41.03 (SD 18.08)	32.67 (SD 9.25)
Camouflage? (yes/no)	51/4	28/2	7/0
Nationality			
British	30	17	4
North American	12	3	1
Western European	7	6	2
Other	6	4	0

Three male participants reported their natal sex as female. All participants who identified their gender as ‘Other’ reported their natal sex as female

### Materials

A newly designed questionnaire of camouflaging was developed by the researchers, in consultation with other experts in ASC, including clinicians, researchers, and adults with ASC. The questionnaire included 23 closed and 20 open questions, and examined participants’ motivations for camouflaging, the characteristics of their camouflaging experiences, the consequences of camouflaging (positive and negative), and their attitudes towards camouflaging (see online Appendix 1). Closed questions were developed from predicted behaviours and observations raised during the development process, although participants were able to give additional detail to their answers if they wished. Open questions were designed to elicit new insights from participants and identify experiences not anticipated by the researchers.

Demographic information about the participants, including details of their ASC diagnosis, was also obtained. Other measures, including those of quality of life, social anxiety, and depressive symptoms, were administered, but not included in the current analysis.

### Procedure

Participants were emailed an online link to ‘a study looking at experiences of coping behaviours in social situations’ (which was hosted by Qualtrics) or followed a link posted on social media. They were reminded that they could withdraw at any point and were under no obligation to answer any question. Participants completed the survey at their leisure and were able to stop and start their responses as they chose, to minimise stress or discomfort from completing the survey.

Early in the questionnaire after demographic data had been ascertained, participants were asked the following question: “Have you ever had the experience of ‘camouflaging’ your autism? A reminder: in this survey we use the term ‘camouflaging’ to refer to ‘coping skills, strategies, and techniques that function to “mask” features of ASC during social situations’.” Those who responded ‘no’ were directed to the end of the questionnaire, where they could leave their thoughts on camouflaging if they wished. These responses were included in the final analysis. Those who responded ‘yes’ completed the full questionnaire. Four females (7% of total number of females) and two males (6% of total males) reported that they had never camouflaged their ASC in social situations. All seven participants who identified their gender as ‘Other’ reported camouflaging their ASC. Responses were saved securely on the Qualtrics server in anonymised format.

Ethical approval for this study was obtained from the University of Cambridge Psychology Research Ethics Committee, reference number Pre.2015.036. Informed consent was obtained from all individual participants included in the study.

### Analysis

Analysis followed the six phases of thematic analysis recommended by Braun and Clarke (2006) with the aim of identifying patterns of information within the data which answered the research questions. This inductive (i.e., data driven) analytic approach was chosen because it does not rely on a rigid theoretical framework for interpretation, and so enables researchers to examine alternative perspectives and identify new information within developing areas of psychology (Willig 2013). Guidelines for good qualitative research (Barker and Pistrang 2005; Elliott et al. 1999; Ritchie et al. 2014) were followed to ensure that interpretations were credible and could be generalised beyond the existing sample. A consensus approach was taken with data extracts read thoroughly by one author (LH) and codes addressing the research questions identified. Initial codes were audited by an independent researcher to confirm that interpretations reflected the data accurately. These codes were then checked by the two senior authors (MCL and WM), and the finalised set of codes was grouped into themes and subthemes. All authors discussed and refined themes until a consensus was reached. Member validation was used as a further credibility check: themes and subthemes were sent to six participants (five female, one male) who had expressed interest in the findings to ensure these accurately reflected their experiences.

## Results

**Fig. 1:**
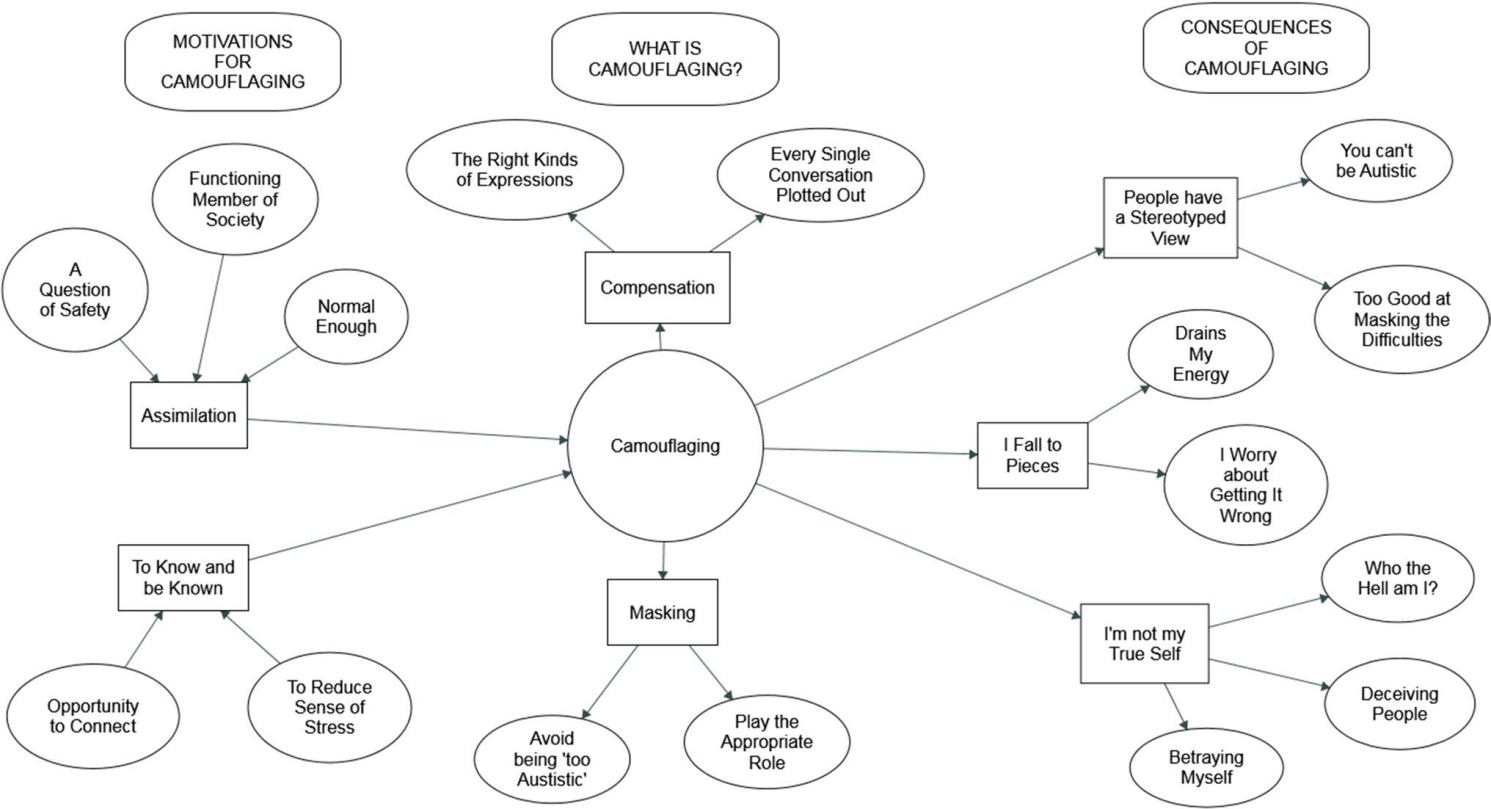
Thematic map of the three stages (motivations, camouflaging, and consequences) of the camouflaging process. Themes are indicated by *rectangles*; subthemes by* ovals*

Seven themes, comprising 16 subthemes, were clustered into three stages of the camouflaging process, as detailed in Fig. 1. Motivations (Assimilation and “To know and be known”) describe the reasons why respondents camouflaged their ASC, including the aims they hoped to achieve as a result. What is Camouflaging? (Masking and Compensation) describes the concept of camouflaging itself, including the techniques used. Finally, the short- and long-term consequences of camouflaging are described through the themes “I fall to pieces”, “People have a stereotyped view”, and “I’m not my true self”. Names of themes and subthemes are taken directly from quotations from respondents. The number of participants who referenced each theme at least once is displayed in Table 2.

**Table 2:** Number of participants who referenced each theme

Theme	Number of participants		
	Female (n = 55)	Male (n = 30)	Other gender (n = 7)
Assimilation: “hide in plain sight”	49	20	7
“To know and be known”	42	24	5
Compensation: “to exceed what nature has given”	45	22	7
Masking: “I’m hiding behind what I want people to see”	38	18	7
“I fall to pieces”	44	21	7
“People have a stereotyped view”	32	6	4
“I’m not my true self”	31	15	3

### Motivations for Camouflaging

#### Assimilation: “Hide in Plain Sight”

Respondents described wanting to camouflage in order to ‘blend in with the normals’. Most respondents reported a social expectation from the general population that individuals with ASC need to change in order to be accepted by others. Respondents’ social and communication difficulties, and their unique behaviours and interests, meant that they stood out from the crowd during social situations. It was felt that the general population viewed this as unacceptable, and so respondents felt a pressure to change their behaviours in order to seem ‘normal enough’.

[I camouflage] to reduce the threat of feeling uncomfortable through being unable to measure up to social expectations. (Male, 62)

I don’t want to draw attention to myself by appearing to be different. (Female, 30)

However, a few respondents suggested that their motivations to camouflage were similar to those of the general population; camouflaging was simply seen as the way in which everyone tries to fit in or hide less desirable aspects of their personality:

Most neurotypicals are camouflaging nearly all the time they are in public. (Male, 79)

A more pragmatic aspect of this motivation was the desire to obtain jobs and qualifications, which respondents felt were less accessible when they were more visibly ‘autistic’. Many respondents described how they would not have achieved as much had they been more open about their ASC characteristics. Camouflaging during these situations was thought to improve employment opportunities, and so enable them to become a ‘functioning member of society’.

I’m pretty sure no-one would ever hire me if I didn’t camouflage in job interviews. (Other, 27)

Camouflaging helps to survive in school and college and it is important for keeping jobs. (Female, 27)

The desire for assimilation was also prompted by concerns for their own safety and wellbeing. Many described being ostracised, verbally or emotionally attacked, and some even reported physical assaults when they had not camouflaged their ASC:

When I was younger and more obviously odd and strange I was thought of as stupid and also badly physically and mentally bullied. I also lost employment. I want to avoid the bullying mostly. I have even been spat at in the street. (Female, 49)

Most attributed this to their perceived differences compared to others, and used camouflaging techniques to minimise these differences and hence reduce the threat. This was particularly the case when describing their experiences in childhood and adolescence; respondents often reported that relations with others improved as they got older and were better able to camouflage their ASC.

If I had known how to camouflage earlier, perhaps I wouldn’t have been such an outcast as a child. (Other, 41)

#### “I Want to Know and Be Known”

The other key motivation for camouflaging was to increase connections and relationships with others. Due to their inherent social difficulties, many respondents reported struggling to make friends and form romantic attachments, despite this being a strong desire. Camouflaging was seen as one way to overcome the initial obstacles to connection and allow for future relationships to develop.

Many respondents wanted to be accepted by others and be able to socialise, but recognised that they lacked the skills needed to make small talk, interact comfortably with strangers, and relax in social situations. This limited their ability to get to know people better. As will be discussed further in the theme ‘Compensation’, camouflaging offers solutions to some of these issues. The payoffs in terms of easier social interaction were a strong motivation for many respondents to camouflage their ASC with others. However, several respondents felt camouflaging was only necessary for the initial stages of a friendship or relationship; once a connection was established, the respondent felt more comfortable showing their ‘true’ ASC characteristics.

I know it is necessary when I am first getting to know someone. After I have known them for a while and they know I have Asperger’s and they are accepting of my quirks, then I can let my guard down more. Connections have to be made initially on neurotypical terms. Then, hopefully, on my terms as well. (Female, 46)

For some, the risk of failure and associated embarrassment created severe anxiety during social interactions; by camouflaging and using structured techniques, respondents could reduce some of this uncertainty and so were more confident in their ability to socialise. Respondents felt that camouflaging would lead to success in a variety of social situations, when compared to their default behaviours or responses.

It enables me to be with other people in a way that is relatively comfortable for me and for them. I avoid looking like a socially clumsy idiot. It avoids the embarrassment and awkwardness of getting things wrong. (Female, 56)

### What is Camouflaging?

#### Masking: “I’m Hiding Behind What I Want People to See”

Masking encompasses the aspects of camouflaging that focus on hiding one’s ASC characteristics and developing different personas or characters to use during social situations. Both of these emphasise a distinction between the respondent’s ‘true’ or ‘automatic’ behaviours, and what they present to the rest of the world.

Camouflaging was partly performed through suppressing, hiding, or otherwise controlling behaviours associated with ASC that were seen as inappropriate in the situation. The extent to which this happened could vary depending on who the person was with; camouflaging tended to occur less often with close friends and family members, although some respondents described camouflaging at all times.

Respondents described attempting to minimise their self-soothing or ‘stimming’ behaviours, and their responses to sensory overstimulation, in order to make their condition less obvious to others. These techniques included using objects as ‘props’ to meet sensory needs in a subtle way, and giving themselves regular excuses to leave overstimulating environments and calm down.

I prevent myself from doing any particularly visible or otherwise noticeable stims: I still find myself doing things like shaking my leg repeatedly without noticing, but don’t make any noises people would think are weird, don’t full-body shake (like with the leg but…all of me), or do any finger movements or tapping etc. that would annoy people. (Female, 20)

Masking enabled respondents to present a different identity to the outside world, one that covered up those parts of themselves they were not happy with. The combination of controlled behaviour and appropriate conversation produced through camouflaging was often described as essential during social interactions, even though this meant concealing one’s actual personality.

I don’t think I’ll ever completely stop wearing the mask. It’s a defence mechanism really. It is easier to have people you’re friendly with, than taking the mask of[f] and revealing the real broken you. (Female, 18)

In some cases, this went as far as portraying an entirely different character, and several respondents likened it to acting or performing a role, complete with costumes. The character or aspects of the role could change across different situations:

I camouflage by putting on a character… I treat my clothes rather like costumes, and certain items of clothing help me to uphold certain personality characteristics of which character I am on that occasion. I have a repertoire of roles for: cafe work, bar work, uni, various groups of friends, etc. They are all me at the core, but they are edited versions of me, designed to not stand out for the ‘wrong’ reasons. (Female, 22)

One way to easily identify the appropriate role to play was to mimic the behaviours of others during a social interaction. Behaviours could be copied directly from the person in front of them, or could be identified and learned from observing others interacting, and even from watching television and films. Some respondents went as far as to copy clothing style, mannerisms, and even interests from others.

I try to copy socially successful people by trying to imitate their speech and body language and trying to understand their interests. (Male, 71)

#### Compensation: “To Exceed What Nature has Given”

The other aspects of camouflaging centre around developing explicit strategies to meet the social and communication gaps resulting from an individual’s ASC, which we call compensation. These camouflaging techniques include specific non-verbal communication strategies and guidelines for successful conversations with others. Respondents often described these techniques as ‘rules’ or expectations from others that had to be met, even if they themselves felt these rules were not necessary.

Explicit, compensatory strategies were reported by many respondents as a vital way to improve non-verbal communication with others. These strategies aimed to help the individual perform behaviours used in typical social encounters, which they would not necessarily perform naturally. Respondents described how these camouflaging techniques required intensive monitoring of the way they presented themselves, in order to ensure they were being performed as correctly as possible.

Forcing and maintaining appropriate eye contact, or attempting to look as close to another’s eyes as possible, was a common compensatory technique reported. Respondents also made an effort to display facial expressions of emotion or interest, even if they didn’t feel this inside. Different expressions were identified as important for different situations, and so many respondents described keeping a mental list of how to behave depending where they were.

I look in people’s eyes when I first meet them/or in formal/professional situations even though I wouldn’t naturally, because I know you’re supposed to. (Female, 26)

I try to look people in the eye and make faces that fit the situation. (Other, 27)

Many respondents noted that their preferred levels of emotional expression and body language did not match those of others around them, and so over-emphasised these behaviours in order to communicate better. This included non-verbal and verbal signs of interest in the interaction, which were also used to encourage others to continue speaking and so take the pressure off the ASC individual to respond appropriately.

My autistic lack of non-verbal signals are read as hostility, arrogance or indifference by people, so I have to act the good will that I genuinely feel. (Female, 45)

I’m not good at knowing when it’s my turn and I also tend to just blurt out things or keep talking when I should have stopped, so I prep myself always in social situations to have a reminder or tag or internal buzzer about not speaking too much and trying to do more listening, nodding, agreeing. (Female, 49)

In addition to these non-verbal techniques, respondents reported developing rules or guidelines to compensate for some of the social difficulties they experienced during conversations. These were more generalised and so could be prepared ahead of time and applied to different situations. These camouflaging strategies were used to help the ASC individual get through ‘small talk’ or more in-depth conversations with minimal stress, and to make the chat more enjoyable for their social partners.

One rule was to ask questions of the other people. Explanations for this varied between respondents, but included minimising the amount of time they had to speak, giving them more time to prepare things to say, and ensuring the ASC individual did not take over the conversation by talking about themselves or their own interests.

I’ve recently tried to institute a rule about asking more “you” questions - how did that make you feel, what did you do next, what do you think about a given thing - instead of “me” or “I” statements. (Male, 29)

My issue is talking too much or saying the wrong things. I tend to think of one or two questions to ask the person and most people are so happy just to talk about themselves that it stops them shining a spotlight on me. I find asking questions is the best deflection and camouflage ever. (Female, 49)

Respondents were often aware that talking only about themselves and their interests was not socially acceptable and so developed strict rules to control their self-focused talk. For some, camouflaging also involved not divulging personal details about themselves, whether to protect themselves from being taken advantage of, or to maintain privacy.

I say as little about myself as possible as the more I say, the more likely it is that I say something inappropriate OR give away too much information about myself which can then be used against me. (Other, 31)

I remain silent when I might otherwise have spoken, knowing that I can’t always tell whether or not my comments would be welcome. I make generic comments rather than offering specific ones that might reveal my more unusual traits. (Male, 29)

Respondents also described spending time before an interaction to prepare topics of conversation, including questions to ask, anecdotes to relate, and potential responses to others. These made them feel more in control of the interaction, and reassured them that they would have structured ‘scripts’ to follow rather than having to spontaneously ‘chat’:

I usually also think up stories and how whole conversations might go before I have them so I have responses practiced as well as potential things to say if the conversation ‘dries up’. (Female, 20)

However, it is important to emphasise that not all respondents developed such structured rules for conversation; some simply had the goal of speaking as little as possible in order to get out of the interaction quickly.

In these social situations, I do not talk about anything of interest to me, I avoid talking much and just pretend to be interested in what people are saying. (Female, 42)

### Consequences of Camouflaging

#### “I Fall to Pieces”

By far the most consistent consequence of camouflaging described by respondents was exhaustion. Camouflaging was frequently described as being mentally, physically, and emotionally draining; requiring intensive concentration, self-control, and management of discomfort. The longer a camouflaging session continued, the harder it became to maintain the intended level of camouflaging. Many respondents reported needing time to recover after camouflaging, where they could be alone and release all of the behaviours they had been suppressing.

It’s exhausting! I feel the need to seek solitude so I can ‘be myself’ and not have to think about how I am perceived by others. (Other, 30)

In addition to this exhaustion, after a camouflaging session was over some respondents would experience extreme anxiety and stress. Respondents felt significant pressure, whether from themselves or others, to camouflage successfully, but many were uncertain of how effective their camouflaging strategies were. Twenty-one respondents (10 male, 11 female) reported being unsuccessful in their camouflaging attempts or reported that they had not achieved the outcomes they intended.

I try to ask them about the things they like, question after question, to keep conversation going but sometimes it doesn’t work and they leave me. (Female, 27)

Camouflaging therefore often involved a constant monitoring of the situation, as if training oneself in self-monitoring, self-awareness, and monitoring others’ reactions, both during and after the interaction occurred, which induced stress and even greater anxiety.

My head will be racing as if I’m interpreting another language. I will be incredibly anxious. It’s like studying for an exam, constantly on edge trying to predict what others will say and do. (Female, 49)

I hate it. I go over and over and over what they said and what I said. Did I understand them correctly, did I respond appropriately, did I make a gaffe? Have I offended anyone? (Female, 45)

In contrast, a minority of respondents reported feeling satisfied and relieved after camouflaging, particularly if they felt as though it went well. For these individuals, camouflaging was rewarding because it enabled them to achieve what they wanted with minimal effort, whether that was getting through a necessary social situation, or being able to make a connection with someone. Interestingly, 60% of those who reported feeling positive or relieved after camouflaging were male (n = 9, compared to six females), in contrast to the majority female total sample.

Small sense of achievement and relief that it is over. (Male, 69)

I am glad that the camouflaging enables me to survive within myself and accomplish any necessary tasks. (Male, 62)

#### “People Have a Stereotyped View”

Many respondents felt that, because their camouflaging changed the way they presented themselves to others, they did not meet the stereotype of ‘an autistic person’ when they camouflaged. In many ways this was construed as positive, since it allowed them to get on in life, succeed in jobs and relationships, and achieve many of the aims they wanted. Some also reported that this enabled them to challenge commonly held views of autism, especially for women. By demonstrating good social skills and educating others about their conditions, respondents hoped to change the public perception of autism and make others more understanding.

People don’t always realise that I have AS, more likely to be socially accepted, more likely to get a job. (Male, 28)

I feel that I’m showing the people I work with that autistic people can have people skills and be good role models (Female, 28)

Some female respondents (n = 7) suggested that others were surprised that they had an ASC, since they differed so much from the public perception of an ASC man with high maths skills, poor eye contact, and uncommon interests.

So many people have a stereotyped view of what ASC looks like. They think people with AS are all geeky, and have little empathy and little insight. They think people with ASC bore on and on about their pet subject and make tactless remarks. They don’t realise that women with ASC tend to internalise things much more and do have empathy and insight, and are very careful not to make hurtful remarks. (Female, 56)

However, there were also negative consequences to not appearing autistic to others. The most striking was that for some respondents their camouflaging, even if it was involuntary, resulted in a delay or questioning of their ASC diagnosis. Respondents reported that parents, teachers, and even clinical professionals refused to believe they could have an ASC, especially if they were female:

The amount of girls that aren’t diagnosed because they are more likely to camouflage than boys is really bad. I went for so long without being diagnosed because they didn’t know that I could pretend to be normal! (Female, 20)

In addition to this, respondents described failing to receive adequate support or allowances for their ASC difficulties, because these difficulties were often hidden behind the mask of camouflaging. Others would therefore give them more responsibilities or expectations than the respondent was comfortable with, because of a perceived level of capability that did not always actually exist.

After beginning graduate school, a lot of issues arose because I was camouflaging to the point that my support needs weren’t being met. So, in that instance, it was detrimental to camouflage. (Female, 24)

I am an SEN teacher and my boss doesn’t know when I am camouflaging. Currently highly stressed because she keeps giving me more work and not realising the stress it is causing. (Female, 44)

For some respondents, this reflected the idea that camouflaging was not a conscious choice; they described wanting to control when and how they camouflage to a greater degree, in order to access support when they needed it:

People need to learn how to drop the camouflage when in situations such as medical assessments or dealing with support professionals otherwise they may be under assessed for support as they appear to be coping. (Female, 28)

For others, however, camouflaging was seen as a deliberate technique to avoid detection. Thus, increasing general awareness of camouflaging strategies by the public, and particularly by employers, was seen as ‘outing’ an ASC individual without their consent. These respondents feared that by giving others the tools to identify their camouflaging, the negative consequences they were trying to avoid would still happen.

If they [employers] can identify camouflaging, then they will “find us out” and reject us. (Female, 68)

#### “I’m Not My True Self”

The final consequence reported by respondents was that camouflaging affected their perception of themselves, in particular how they represented themselves to the outside world and their sense of authenticity. For many respondents, by camouflaging their ‘true’ or natural behaviours they were lying about who they were. This was often regretted by the respondents, who wanted to be happy as they were, but felt that the pressures of the typical social world meant this was not possible.

I don’t care about being different, I like my differences (apart from things feeling really stressful and no confidence) but I don’t want to deal with peoples’ negative and sometimes evil reactions. I feel like the weight of a black cloud is hanging on me having to be this fake version of me. (Female, 48)

In an extension of this, for some respondents their camouflaging behaviours contradicted the important role they attributed to ASC in shaping their identity. Despite feeling proud of their ASC diagnosis, and the community they were a part of, they still deliberately camouflaged the behaviours associated with this diagnosis. These individuals felt that by hiding their ASC characteristics, they were betraying the ASC community as a whole.

It’s mentally exhausting constantly having to be something else, literally never being able to be myself, and kind of sad too I guess? I even stop myself doing certain tics and things automatically when I’m by myself and that kinda sucks, that I’m not even me on my own. I guess I’m letting down the side a bit by hiding my autism; I am very vocal about stigmas and stereotypes with mental illness, and do talk about my anxiety openly, so I don’t know why autism is different. (Female, 20)

Some respondents felt that the relationships they formed through camouflaging were based on deception, and therefore the relationships themselves were false. This reinforced experiences of loneliness and isolation, as they felt no one truly knew them or understood them. Some also felt bad for deceiving their friends and even loved ones.

I feel sad because I feel like I haven’t really related to the other people. It becomes very isolating because even when I’m with other people I feel like I’ve just been playing a part. (Female, 30)

I was married for 15 years and was camouflaging in high gear during that time… My husband would occasionally say to me that he wondered if I was really who I was. I think he would get glimpses of the real me. I didn’t even know who the real me was… The marriage ended in divorce. (Female, 64)

The situations in which respondents camouflaged were so extensive for some, they felt that they were losing sense of who they truly were. Respondents often felt they were playing so many different roles, it was hard to keep track of their authentic sense of identity. This increased the anxiety and stress associated with camouflaging, as individuals lost a sense of grounding and security in who they were.

Sometimes, when I have had to do a lot of camouflaging in a high stress environment, I feel as though I’ve lost track of who I really am, and that my actual self is floating somewhere above me like a balloon. (Female, 22)

## Discussion

This study identified key themes underlying the motivations, techniques, and consequences associated with social camouflaging amongst adults with ASC. The vast majority of participants (male, female, and of other genders) reported camouflaging to some degree, although there was significant variation in individual experiences of camouflaging. The results were combined into a model of the camouflaging process, which we hope will contribute to the generation of testable hypotheses and identification of avenues for future research.

The themes revealed two key motivations for camouflaging; assimilation and connection. This suggests that camouflaging behaviours come from multiple sources. They may be internally driven by the individual to accomplish specific goals such as friendships, but they may also be produced as a response to external demands placed on how a person should behave in society. The differential influence of each of these motivations varies between individuals, but our findings suggest that people are strongly motivated by wanting to avoid discrimination and negative responses from others. This conclusion is supported by a recent study demonstrating that non-autistic individuals judge autistic people more negatively, and are less willing to interact with them, even after only brief exposure to the autistic individual (Sasson et al. 2017). Several participants suggested that improved education and acceptance of ASC amongst the general public would improve their social experiences significantly, and would allow them to both fit in and increase their connections without the need to camouflage.

Respondents described a wide variety of techniques used as part of their camouflaging behaviours, and further research is needed to determine the extent to which specific techniques can be generalised to all people who camouflage. The two main themes found here, masking and compensation, appear to relate to the motivations of fitting in and forming connections respectively; respondents used techniques to mask their ASC in order to appear like other people around them, and compensated for their social communication difficulties in order to make better connections with others. However, it remains to be seen whether these two goals of camouflaging are entirely separate, or whether the same techniques can be used to further both aims.

There was extensive variation in the consequences of camouflaging reported, but one of the most striking findings was that the vast majority of participants reported some unpleasant and unwanted consequences of camouflaging. These included the exhaustion experienced during and after camouflaging, which has been identified in previous research (Tierney et al. 2016). Our findings suggest that, if people with ASC want to continue camouflaging in the ways reported in our study, those supporting them should be aware of the associated strains. Time alone to recover was identified as an important tool to help participants continue camouflaging, and could be utilised by employers and schools to make these environments more accessible for ASC individuals.

In addition, a profound consequence of camouflaging was a change in self-perceptions, as detailed by the theme ‘I’m not my true self’. Camouflaging appears to challenge many participants’ views towards themselves, and produce negative emotions and attitudes, such as being a ‘fake’ or losing their identity. It may be that the rigidity of thinking and scrupulous honesty that are present in many individuals with ASC leads them to view any change in self-presentation as false (Chevallier et al. 2012). Regular camouflaging would consequently increase the individual’s perception of themselves as a ‘liar’ or inauthentic person, and could lead to long-term negative impacts on self-esteem. This could account for the finding that some participants viewed camouflaging as lying, in contrast to those who viewed it as a performance.

We can only speculate whether differences in participants’ attitudes towards camouflaging, including the motivations and techniques used, may lead to differences in the consequences of camouflaging. Interestingly, positive consequences were reported more frequently by males than females or those of other genders. This could suggest that camouflaging is more likely to be a satisfying process for males with ASC given present gendered social-cultural contexts; alternatively, it may reflect gender differences in the actual camouflaging techniques used, which produce different consequences. However, some participants reported that their camouflaging strategies were not always performed successfully; a relatively large proportion of these participants were male, in contrast to the gender ratio of the overall sample. There may be a discrepancy between desire to camouflage and ability to do so, and this too should be investigated in different genders and across the entire autism spectrum. The potential gender difference corresponds well with a recent study showing on-average lower level of camouflaging and stronger association between camouflaging and depressive symptoms (i.e. the more camouflaging, the higher level of depression) in men with ASC, compared to women with ASC (Lai et al. 2016). It may be that females with ASC who camouflage tend to do so more successfully than males.

Previous researchers have suggested that camouflaging by females with ASC might account for the gender disparity in diagnosis (Gould and Ashton-Smith 2011; Kreiser and White 2014; Lai et al. 2015). Our study is not designed to directly test this idea, or to compare the extent of camouflaging between different groups. We found that relatively equal numbers of males and females, and all individuals of other genders, reported camouflaging, and no consistent patterns of differences in camouflaging behaviours between males and females were identified. However, some female and other-gender participants argued that camouflaging was a specific reason for their own or others’ late diagnosis, suggesting that society places higher demands on social ability and assimilation for people perceived as female. Indeed, a recent study in elementary school children shows that the gendered, female social landscape supports ASC girls for camouflaging (e.g., staying in close proximity to peers) and therefore if clinicians and teachers rely on a male landscape to detect ASC characteristics (e.g., social isolation on the playground), females will tend to be left unidentified (Dean et al. 2016). Further examination of the impact of camouflaging behaviours in all genders is essential to understand the difficulties in accessing support by those who do not show a ‘typical’ ASC presentation.

One explanation for the similarities in camouflaging between males and females found here is that our sample was self-selecting, in response to a call for participants for ‘a study looking at experiences of coping behaviours in social situations’. Although previous experience of camouflaging was not required to take part in the study, potential participants might have interpreted the advertisement in this way. It is therefore possible that our sample comprised only those people who had experienced camouflaging, which might include a substantial number of ASC females, but a smaller proportion of ASC males. The majority of those who did not take part, because they had never or only rarely experienced camouflaging, may have more likely been male. This would account for the high proportion of female participants in our study, in contrast to previous research into ASC. Further investigation of camouflaging behaviours across the entire ASC population would shed more light on this.

An alternative explanation is that camouflaging is equally common in males and females with ASC. Previous research has either theorised that camouflaging is more common in females (Lai et al. 2011; Wing 1981), has only included female samples (Bargiela et al. 2016; Tierney et al. 2016), or has observed on-average more evident camouflaging in females than males (Dean et al. 2016; Lai et al. 2016). If camouflaging does indeed lead to not receiving the diagnosis, there may, in fact, be a significant number of both males and females with ASC missing out on the support they might need. Future research could test this possibility by comparing camouflaging levels in males and females with high ASC traits, but who have not received an ASC diagnosis. However, this also leads to a point that was raised by some of the participants who reported not camouflaging—the concept that if people are camouflaging so successfully that they are not diagnosed, they may not need a diagnosis or related support. While this may seem plausible to those who view camouflaging as a successful, low-impact strategy, the significant difficulties and uncertainty reported by our participants tell us that people who camouflage still need to be able to access appropriate support.

This issue reflects a concern voiced by some participants, viz. that increasing the awareness of camouflaging in the general public might actually lead to worse outcomes for some individuals with ASC. Those participants who used camouflaging to hide their ASC, especially at work, often viewed their camouflaging as a defensive strategy protecting them from discrimination. They worried that if other people were able to identify camouflaging, the ASC individual might lose this protection and be treated unfairly. It remains to be seen how much camouflaging in ASC can be identified by others. Many participants felt their camouflaging was at times unsuccessful, or reported occasions where another person had commented on their techniques. This concern suggests that research and public education regarding camouflaging needs to be performed in consultation with a range of people from the ASC community to ensure that increasing information helps rather than harms. More crucially, this concern voiced by some participants once again emphasises that the outcome of individuals with ASC does not solely rely on personal characteristics—it can more fundamentally rely on how the social contexts treat them. A better person-environment fit is the key, and this involves ‘treating the environment’ to reduce stigmatization attached to autism and barriers to social life (Lai and Baron-Cohen 2015).

### Strengths and Limitations

One strength of this study was the high proportion of females and those of non-binary gender, many of whom were diagnosed later in life. This is an under-represented population, and it is important to include their voices and insights, which may be different to those of the majority male, younger samples included in previous research. However, because of this our sample was not fully representative of the entire ASC community. Intellectual ability was not measured, although it can be assumed that participants should have had close to or average cognitive abilities in order to be able to complete the online, text-based survey. The cognitive and self-reflecting abilities required to complete the survey may also mean that our sample were better able to perform successful camouflaging behaviours than others on the autism spectrum.

As a result, our findings cannot be said to represent the views of those with ASC who also have intellectual disability, or who cannot express themselves in written English. Developing more accessible measures of camouflaging, such as self-report questionnaires that can be orally or visually administered, or measures to identify camouflaging behaviours, would improve our ability to understand camouflaging across the whole ASC community. This study was not designed to measure camouflaging behaviours across the ASC population, but to identify the component parts of the construct of camouflaging. We hope that with these results, future research can investigate the functional and demographic characteristics of those individuals with ASC who do or do not camouflage, including those with non-binary gender identities and/or gender dysphoria, characteristics that may also contribute to the need for camouflaging, and should be explored in their own right. Larger and more varied samples of individuals from across the autism spectrum should be included to further refine our understanding of camouflaging in the future.

As previously mentioned, our sample only included adults with a confirmed diagnosis of ASC; it is therefore possible that those who are most likely to camouflage were not included in our study as they would not have met the diagnostic criteria. A typically developing comparison group was not included in this study due to the difficulty of operationalising camouflaging for individuals with limited ASC-related characteristics. However, several participants reported having camouflaged for years before receiving a diagnosis later in life, suggesting that our findings have relevance for undiagnosed ASC individuals. Using the behaviours and themes identified in this study, descriptions of camouflaging suitable for the general population can now be developed. Future research in individuals with high levels of ASC traits, regardless of their diagnosis, may reveal more about how camouflaging varies between those who do and do not receive an ASC diagnosis. In addition, further qualitative and quantitative research comparing the camouflaging experiences of individuals from different age groups may reveal more about how camouflaging develops and changes across the lifespan.

The inductive nature of this study has resulted in novel avenues for research, such as focusing on the impact of camouflaging on identity, which may not have otherwise been considered. In addition, although camouflaging has previously been described as mainly a female expression of ASC, we found that many males and individuals of other genders also reported camouflaging. A recent study operationalising camouflaging using existing ASC-related measures also shows wide variability of the level of camouflaging in both men and women with ASC, indicating that camouflaging is not a female-specific phenomenon (Lai et al. 2016). In the present study, no statistically tested gender differences in camouflaging behaviours or outcomes were presented due to the qualitative nature of the data, and no analysis of the subjective or objective success of camouflaging attempts was made. However, our findings have produced the first known conceptual model of camouflaging, with key themes and components as identified by individuals who camouflage. We hope that future research in this area will use the themes identified here to develop precise, testable hypotheses for qualitative or quantitative research into camouflaging and the sex- and gender-informed phenotypes of ASC.

The next stage of research requires the development of measures of camouflaging behaviours, in order to standardise and compare camouflaging experiences between autistic and non-autistic individuals and allow for follow-up quantitative research. We hope that the model presented in this paper, and in particular the behaviours described in the ‘masking’ and ‘compensation’ themes, will provide a framework for the development of such a measure. Furthermore, studies delineating component psychological constructs and interpersonal-contextual processes underlying the themes identified here will deepen our understanding of the mechanisms underlying camouflaging. Eventually this may lead to novel support strategies and advocacy that maximise the positive consequences and minimise the negative consequences of camouflaging—and to attain the most appropriate person-environment fit for each individual with ASC.

## Conclusions

This study demonstrates that camouflaging of ASC-related characteristics in social situations may be a common behaviour amongst adults with ASC. Camouflaging is motivated by the desire to fit in with others and to make connections. The behaviours themselves can be grouped into masking and compensation strategies. In the short term, camouflaging results in extreme exhaustion and anxiety; although the aims of camouflaging are often achieved, in the long-term there are also severe negative consequences affecting individuals’ mental health, self-perception, and access to support. Our findings demonstrate that camouflaging is an important aspect in the lives of many individuals with ASC. Future research is needed to quantitatively measure camouflaging and compare techniques in individuals with ASC of all genders, to identify demographic and ASC characteristics associated with individual variation in camouflaging and its outcomes, to uncover underlying psychological and interpersonal/contextual processes, and to devise strategies that minimise negative impacts of camouflaging and facilitate the realization of maximal individual potential.

## Electronic supplementary material

Below is the link to the electronic supplementary material.

Supplementary material 1 (DOCX 16 KB)

## Footnotes

1 We use the term ‘Autism Spectrum Condition’ (ASC) when referring to a diagnosis of Autism Spectrum Disorder, to be respectful to those on the spectrum who feel that the term ‘disorder’ is stigmatising, whereas ASC is compatible with the presence of both the strengths and difficulties of people on the spectrum.
