# Supplementary material for: “Putting on My Best Normal”: Social Camouflaging in Adults with Autism Spectrum Conditions
Source: J Autism Dev Disord. 2017 May 19;47(8):2519–34. doi: 10.1007/s10803-017-3166-5 (PMC5509825; doi:10.1007/s10803-017-3166-5)
Supplement: Supplementary file 1 — Supplementary material 1 (DOCX 16 KB) [file 10803_2017_3166_MOESM1_ESM.docx]

**Appendix 1**

**Camouflaging Questionnaire**

*NB: Closed questions have response options below.*

1. Have you ever had the experience of 'camouflaging' your autism? A reminder: in this survey we use the term 'camouflaging' to refer to 'coping skills, strategies, and techniques that function to "mask" features of ASC' during social situations.

Yes/No

1. In what situations do you camouflage (for example, when meeting new people, in large groups, job interviews etc.)?
2. How frequently do you camouflage in social situations? Please give more detail if you would like.

| Always (camouflage in almost all social situations [>95%]) | |
| --- | --- |
| Often (camouflage in most social situations [>50%]) |  |
| Sometimes (occasionally camouflage in social situations [<50%]) |  |
| Never (do not camouflage in social situations [0%]) |  |
| None of the above (please describe) |  |

1. In social situations, how do you camouflage/what do you do when you camouflage? Please provide details and examples, for instance the behaviours and thoughts you experience.

Note: we would like you to share your own personal experiences about what you do, without presuming what these might be, so we have not listed any examples of common behaviours here. However, the next section of questions will ask you directly about examples we know from others’ experiences to date.

Some people with ASC have reported the following as examples of camouflaging/coping in social situations. We would be interested to know if you have ever:

*Responses for questions 5-22:*

| Always | Often | Sometimes | Never |  |
| --- | --- | --- | --- | --- |
| 1. Tried to hide difficulties with eye contact by pretending that you are making eye contact? | | | | |
| 1. Tried to hide your social stress by pretending that you are relaxed? | | | | |
| 1. Tried to use learned/pre-prepared jokes or one-liners in conversation? | | | | |
| 1. Tried to keep conversation flowing by talking about your special interests? | | | | |
| 1. Tried to make your movements more natural by deliberately practicing them when alone? | | | | |
| 1. Tried to copy other people's body language? | | | | |
| 1. Practiced how to make the intonation of your voice sound more 'normal', by speaking aloud to yourself when you are alone? | | | | |
| 1. Practiced facial expressions in front of the mirror when you are alone? | | | | |
| 1. Tried to learn to use gestures to blend in amongst neurotypicals? | | | | |
| 1. Pretended to be interested in topics during conversation with others, just to fit in? | | | | |
| 1. Engaged in other (non-social) activities (e.g. studying objects in the situation, cleaning, using smartphone/tablet) in social situations? | | | | |
| 1. Forced yourself to approach strangers to increase your confidence in social interaction? | | | | |
| 1. Studied people's behaviour in films by playing certain clips over and over again, and then tried to imitate these? | | | | |
| 1. Observed one particular person in your class (when you were a student), workplace, or neighbourhood, who was socially successful, and tried to copy his/her mannerisms, talking style, dress style etc.? | | | | |
| 1. Consciously acted in social situations? | | | | |
| 1. Conducted 'research' (e.g. reading novels, learning about psychology) to work out the rules of human behaviour? | | | | |

1. Tried to create rules to guide you through a social interaction?

21a. Please tell us your rules:

1. Tried to develop rules that you follow to keep conversation going, such as 'talk a lot', 'tell the listener an anecdote about something that happened', 'keep talking so that the conversation doesn't dry up' etc.?

22a. Please tell use your rules for keeping conversation going (for example, thinking about how best to imitate neurotypical people):

1. In these behaviours and thoughts that you have when you camouflage, which ones do you need to 'perform' / 'act' with effort?
2. In these behaviours and thoughts that you have when you camouflage, which ones are more 'intuitive' / 'automatic'?
3. Why do you camouflage during social situations?
4. To what degree do you think camouflaging is necessary for you in social situations?
5. How do you know if your camouflaging works or not?
6. How successful do you think your camouflaging is? Please give more detail if you would like.

| 1 (not good at all) |
| --- |
| 2 (fair) |
| 3 (mostly good) |
| 4 (very good) |
| X (unsure/not known) |

1. Has anyone told you that they have noticed that you are camouflaging?

Yes/No

29a. How did they recognise that you were camouflaging?

1. How do you feel after camouflaging?
2. When you get home after a day of work/school, or an episode of 'pretending' to be neurotypical, do you feel exhausted and in need of isolation?

Yes/No

1. At what age did you consciously start to camouflage in social situations?
2. What are the skills that are needed for successful camouflaging?
3. How did you learn to camouflage?
4. What are the negative consequences of camouflaging for you?
5. What are the positive consequences of camouflaging for you?
6. Have you ever decided not to camouflage in social situations? Why?
7. Do you think support programs for individuals with an ASC should cover the topic of camouflaging (for example, when and how to do it, knowing its consequences)? Why?
8. Do you think people working with individuals with an ASC (for example, employers, clinicians, teachers etc.) should be knowledgeable about, and be able to identify, camouflaging? Why?
9. Do you think 'camouflaging' is the right word to describe what you may do in social situations? If not, what do you think is a better term to call it? Why?
